# HPLC methods for purity evaluation of man-made single-stranded RNAs

**DOI:** 10.1038/s41598-018-37642-z

**Published:** 2019-01-31

**Authors:** Anastassia Kanavarioti

**Affiliations:** Yenos Analytical LLC, 4659 Golden Foothill Pkwy, Suite 101, El Dorado Hills, CA 95762 USA

## Abstract

Synthetic RNA oligos exhibit purity decreasing as a function of length, because the efficiency of the total synthesis is the numerical product of the individual step efficiencies, typically below 98%. Analytical methods for RNAs up to the 60 nucleotides (nt) have been reported, but they fall short for purity evaluation of 100nt long, used as single guide RNA (sgRNA) in CRISPR technology, and promoted as pharmaceuticals. In an attempt to exploit a single HPLC method and obtain both identity as well as purity, ion-pair reversed-phase chromatography (IP-RP) at high temperature in the presence of an organic cosolvent is the current analytical strategy. Here we report that IP-RP is less suitable compared to the conventional ion-exchange (IEX) for analysis of 100nt RNAs. We demonstrate the relative stability of RNA in the denaturing/basic IEX mobile phase, lay out a protocol to determine the on-the-column stability of any RNA, and establish the applicability of this method for quality testing of sgRNA, tRNA, and mRNA. Unless well resolving HPLC methods are used for batch-to-batch evaluation of man-made RNAs, process development will remain shortsighted, and observed off-target effects *in-vitro* or *in-vivo* may be partially related to low purity and the presence of shorter sequences.

## Introduction

The discovery of Chromatography, i.e. separation of a mixture into its components, approximately 120 years ago is credited to Mikhail Tsvet, a Russian-Italian botanist. A major revolutionary step in chromatography was the advent of high-performance liquid chromatography (HPLC) instruments invented 50 years ago^1,2^. In HPLC, a liquid mobile phase (MP) carries a mixture of compounds through a column packed with particles. As a pump forces the MP through the column, the components of the mixture interact with the stationary phase - the particle’s surface - to different degrees and are separated in the process. HPLC’s first application was the resolution of nucleic acids exploiting IEX normal mode^3^, even though this type of chromatography was later abandoned. Since then a steady improvement in instrumentation and column packings have yielded methods for analysis and purification of both synthetic materials as well as compounds from biological fluids^4–7^. HPLC remains the most widely used analytical technology especially for purity determination and batch-to-batch comparison of compounds poised for pharmaceutical use^8^. Most chromatography suppliers claim applications for oligonucleotide analysis, but in reality good analytical methods exist for up to 25nt^9,10^ and become less optimal as the length increases^5,6^.

The synthesis as well as the purification of oligoribonucleotides are intrinsically less efficient compared to the synthesis and purification of their deoxy counterparts, primarily due to the presence of the 2′-OH that needs to be protected during synthesis^11–16^. Protection of 2′-OH and deprotection at the end of the synthesis may not be accomplished at 100% yield, so the overall efficiency of RNA synthesis is compromised compared to DNA. Coupling efficiency in oligonucleotide manufacturing refers to the success rate of a synthesizer adding a new base to a growing nucleic acid chain. This measure is especially important for long oligos, because as the length of an oligo increases, small differences in coupling efficiency have dramatic effects on the yield of the full-length product. Here are two examples to illustrate the compounding effect of less than 100% efficiency in the synthesis of a 100nt vs the synthesis of a 50nt. With an average 99.0% efficiency at each step, the calculated fraction of the 100nt in the final product is 0.366, whereas the calculated fraction of the 50nt is 0.605, almost double. With an average 98.0% efficiency at each step (just 1.0% less), the corresponding calculated fraction for a 100nt is 0.13 and for a 50nt is 0.36. The “theoretical” 13% yield to make a 100nt with an 98.0% average step efficiency is consistent with literature claiming 5.5% yield, after purification, for the optimized synthesis of a 110nt RNA oligo^17^. With synthetic oligos the highest HPLC peak is very likely to correspond to the desired oligo. Identification of the final product is conducted using IP-RP HPLC analysis directly followed by mass spectrometric (MS) detection^18^. It should be noted that product identification by mass determination does not require resolution, and therefore identification is independent of relative abundance. However the effectiveness of purification/isolation of the desired product correlates with the product’s abundance in the crude mixture^5,6^.

In addition to the synthetic efficiency issue, the longer oligoribonucleotides, let us say longer than the 50nt, exhibit self-structure within the otherwise linear polymer^19,20^. Such self-structure could be due to short intramolecular double stranded regions between distant sequences, to regional stem-loop folding, to Hoogsteen base-pairs in G-rich sequences, and/or to a wide array of base-stacked conformations. Self-structure leads to the presence of several conformers of comparable stability. Conformers may bear scientific interest regarding *in-vitro* and *in-vivo* activity, but they are an additional obstacle to good separation of the mixture into the desired oligo and its impurities. Hence analysis of longer RNAs necessitates denaturing conditions. Such conditions are (i) aqueous MP at pH 12 (see later), (ii) high temperature, and (iii) the presence of an organic cosolvent, such as methanol (CH_3_OH) or acetonitrile (CH_3_CN), in the MP. Additional additives are formamide and urea, favored by molecular biologists, but not by HPLC analysts. All these additives/conditions act by disrupting base-pairing and base-stacking interactions, and therefore practically linearize the nucleic acid. Linear, non-structured, polymers may elute as sharp peaks by HPLC, and thus resolve from closely related impurities.

Credit for the development of column packings and methods to resolve oligonucleotide mixtures, in this author’s opinion, should be given to the late Dr. Leslie E. Orgel of the Salk Institute and his coworkers for the research they spearheaded in support of the “RNA world” hypothesis^21–23^ (first described by Alexander Rich in 1962, and later coined by Walter Gilbert in 1968). In the 1970s-80s the primary mode of chromatography was normal phase^3,24^, eventually completely replaced by reversed-phase. In the 1970s Orgel and coworkers discovered the non-enzymatic, template-directed synthesis of oligoribonucleotides^25^. Using phosphoimidazolide activated ribomononucleotides, as building blocks, and homo- or hetero-polymeric nucleic acids, as templates, they demonstrated formation of the complementary strand as a series of oligos up to the 40nt^26^. In order to investigate length and linkage, they took Kel-F packing, coated it non-covalently with Adogen, a mixture of tetraalkylammonium compounds, and packed it in HPLC columns (named RPC5)^27,28^. This was the first packing known to resolve oligos up to the 40nt based on length and internucleotide bond linkage (2′-5′, 3′-5′ as well as pyrophosphate)^26^. For the next two decades oligonucleotide analysis was accomplished using packing material from Orgel’s Laboratory, and a MP made out of aqueous 10 mM NaOH (pH 12.0, see also Results below) and NaClO_4_ for salt gradient elution^29–31^. The RPC5 packing was not user-friendly, and ultimately replaced by commercially available ones, that barely claim to match its resolving power.

Several HPLC interaction modes have been used successfully for the separation of an oligonucleotide mixture. While initially the mode of choice was normal phase IEX, in 1987 we introduced reversed-phase mode for separation of oligos with N < 5^32^. Since nucleic acids are easily retained on packings covalently modified with tri- or tetra-alkylammonium substituents, IEX methods became popular. In IEX increased salt concentration in the MP promotes elution of oligo of length N ahead of oligo with length N + 1, simply because oligo N is retained less strongly than oligo N + 1. The addition of a volatile ion-pairing agent, such as triethylamine acetate buffer (TEAA), in the MP of a reversed-phase packing facilitates both elution from the column and good separation^9,18^. The latter, IP-RP, quickly became a favorite, as it allows for leading the LC flow directly onto a mass detector, and enables MS determination for every HPLC peak. Other modes, like size-exclusion, affinity, and hydrophobic interaction have also been used in special cases. State-of-the-art in RNA oligo separations is currently up to the 60nt using capillary gel electrophoresis (CGE)^33^, or HPLC in conjunction with IP-RP^18,34–36^ or with IEX modes^37,38^. Limited number of companies and research groups^17^ claim synthesis, analysis, identification, and purification of RNA oligos at the 100nt level. Transfer RNAs (tRNAs) are about 76nt long and known to be the most self-structured RNAs, typically found in a stable cloverleaf conformation^39^. Research in proteomics has led to large-scale, cell-free use of *in-vitro* transcribed (IVT) tRNAs as carriers of both native and non-native amino acids to synthesize novel proteins^40^. While analysis/purification of native as well as IVT tRNA is laborious and presents challenges, the IEX HPLC method developed here may provide purity evaluation and serve as a purification method (see Results).

Until the discovery and commercialization of Clustered Regularly Interspaced Short Palindromic Repeats (CRISPR) as a technology with therapeutic potential for human diseases, HPLC analytical methods for RNA oligos up the 60nt were adequate for most applications. CRISPR is a family of DNA sequences in bacteria that contain sequences from viruses that have attacked the bacterium. These sequences play a key role in a bacterial defense system, and form the basis of a genome editing technology known as CRISPR/Cas9 that allows for genome modification (addition or deletion) within any organism, including humans. A number of companies are currently actively promoting CRISPR technologies for several diseases, and plan to submit to the FDA Investigational Drug Applications (IND) for Cas9 protein, messenger RNA (mRNA) responsible for Cas9 protein synthesis, and sgRNA with sequence complementary to the gene that is to be cut out or replaced. sgRNAs are typically 100nt long and analytical methods for RNAs of such length are sparse^17^. In this report we illustrate that IP-RP may be less optimal for purity evaluation of sgRNA compared to conventional IEX. We then develop a fully denaturing basic IEX HPLC method for purity evaluation of sgRNA, and demonstrate that such method is also suitable for tRNA and mRNA. Most importantly, we design and illustrate protocols to use with any suitable IEX column and any RNA sample to assess on-the-column and off-the-column stability.

## Results and Discussion

### Selection of HPLC columns and materials

Method development efforts centered around the DNAPAC PA200, 8 μ bead size, (abbreviated DNAPacIEX) form ThermoFisher Scientific (earlier Dionex) as IEX HPLC column^41^. Earlier method development in our own Laboratory comparing a number of HPLC columns from different suppliers showed base-line resolution among a series of 20nt long oligodeoxyadenylates where the 11th base was replaced by a different nucleotide than dA^42^. Literature illustrating N + 1 resolution of synthetic 60nt oligos^41^ led us to the conjecture that DNAPacIEX may exhibit the highest possible separation for oligos with N > 60. Some of the advantages of the DNAPacIEX are: usability in a pH range of 2.5 < pH < 12.5, in a temperature range up to 85 °C, and the compatibility with MeOH or CH_3_CN^41^. There is actually a newer version of the DNAPAC PA200 with a 4 μ bead size, and consequently of better resolution, but it exerts higher back pressure (>450bars) compared to what our HPLC instrument can handle, and therefore was not tested here. As IP-RP HPLC column we selected one from the same supplier, DNAPAC RP (abbreviated DNAPacRP)^34^, with the reasoning that any IP-RP HPLC column produced by this specific supplier would have been compared side-by-side with the DNAPacIEX before entering the market place, and therefore judged to be, at least, as resolving as the DNAPacIEX. Indeed literature for DNAPacRP illustrates separation of synthetic 60nt RNA oligos with N + 1 resolution^34^. As test articles we used two 32nt RNA oligos differing by 4 nucleotides, a canonical 100nt RNA and the 94nt truncated RNA with the same sequence as the 100nt, but missing 6nt at the 3′end, and a heavily methylated 100nt RNA of the identical sequence as the canonical 100nt, but with 46 2′OMe groups. In addition we tested tRNA (*E. Coli*) and two mRNAs, EGFP with 996nt and Cas9 with 4500nt. The 100nt RNAs serve as representative examples for sgRNA. Suppliers and RNA sequences can be found in the Materials and Methods section.

### RNA’s secondary structure is an obstacle to purity determination

**Figure 1:**
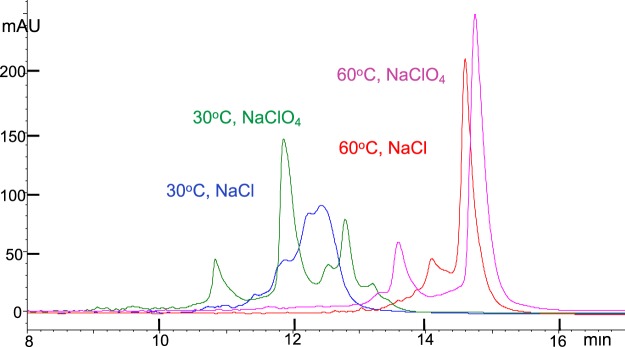
DNAPacIEX HPLC profiles of a 100nt RNA(2′OH) analyzed in neutral pH; increasing temperature results in fewer peaks attributed to diminished secondary structure. Blue and red traces with NaCl, green and pink traces with NaClO_4_ elution; blue and green traces with HPLC column compartment at 30 °C, red and pink traces at 60 °C. Flow, 0.9 mL/min; NaCl gradient elution in 15 min from 20% to 70% of 1.5 M NaCl in 25 mM TRIS.HCl pH 7.0. NaClO_4_ gradient elution in 15 min from 10% to 50% of 0.5 M NaClO_4_ in 25 mM TRIS.HCl pH 8.0.

HPLC analyses of the two 32nt RNAs (abbreviated oligo1 and oligo2, see sequences in Materials and Methods section) using DNAPacIEX in MP with pH in the range of 7 < pH < 12 exhibit sharp peaks and comparable impurity profiles, simply because these two RNAs are short and devoid of secondary structure. On the contrary, a 100nt RNA with 46 2′-OMe groups, abbreviated RNA(2′OMe), see sequence in Materials and Methods, exploited here as an example of a sgRNA, exhibited a complex HPLC profile upon analysis in a pH 7 aqueous buffered MP, reminiscent of size exclusion chromatography of a protein mixture and discordant with the profile of a synthetic oligo. Similarly, analysis of the RNA(2′OMe) by Capillary Zone Electrophoresis (CZE) at pH 9.3 in borate buffer exhibits a very broad peak. CZE does not have the capability to resolve a mixture of similar oligos, and should produce a single sharp peak. A single sharp peak by CZE was only observed after incubating the sample at 95 °C for 5 min, known to disrupt secondary structure, cooling it down to room temperature (RT) instantaneously, and analyzing it within minutes. We hypothesized that the secondary structure was primarily the result of the heavy methylation, so we tested the unmethylated 100nt RNA(2′OH), same sequence as the RNA(2′OMe), only to observe a comparable HPLC profile (see Fig. 1). By increasing the temperature of the HPLC column compartment from 30 °C to 60 °C, the DNAPacIEX HPLC profile became more defined, i.e. included fewer main peaks (Fig. 1) strongly suggesting that the secondary structure is the result of the sequence and the length, and not the methylation. Analysis of RNA(2′OMe) by IEX at 60 °C, using 100 mM of sodium carbonate buffer at pH 11.0 and increasing salt (NaCl or NaClO_4_) for elution, led to the observation of two well resolved peaks. These observations in conjunction with the experiments using IP-RP (see next section and Supplementary Information) led to the conclusion that there are, at least, two stable conformers associated with this specific sequence, one of them being the random coil, the only one stable at the highest temperature.

### Denaturing conditions for RNA analysis

**Figure 2:**
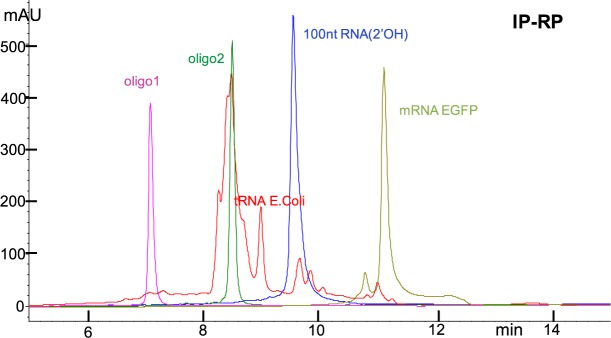
DNAPacRP HPLC profiles of a series of RNAs analyzed with the same method at 65 °C with CH_3_CN as cosolvent. MPA, 0.1 M TEAA pH 7.0 in water; MPB, 25–75% CH_3_CN-H_2_O (v/v) in MPA; gradient 15% to 95% MPB in 16 min, flow at 0.35 mL/min. tRNA *E. Coli* exhibits a major peak coeluting with oligo2, a 32nt RNA (see Materials and Methods), while the rest of the material elutes between 7 and 11 min. Coelution of a major component in tRNA *E. Coli* with a 32nt oligo is inconsistent with tRNA composition. 100nt RNA(2′OMe) elutes after 100nt RNA(2′OH), but 100nt RNA(2′OH) and the truncated 94nt RNA(2′OH) do not resolve in this chromatography, as tested separately and in a mixture (see Fig. S2 in Supplementary Information). mRNA EGFP appears impure by IP-RP and by IEX analysis (see Fig. 3).

**Figure 3:**
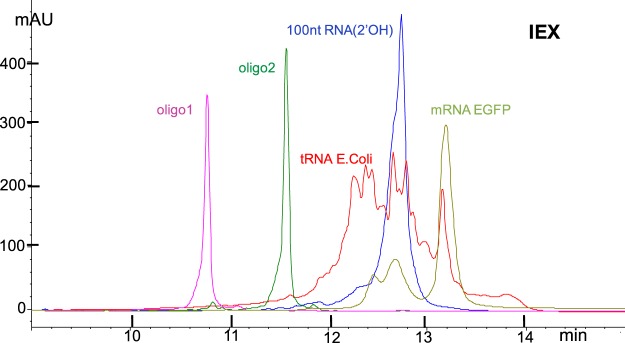
DNAPacIEX HPLC profiles of a series of RNAs analyzed with a pH 12 MP and NaCl as salt gradient at 10 °C. MPA, 10 mM NaOH in water at pH 11.9 ± 0.3, MPB, 1.5 M NaCl in MPA at pH 11.9 ± 0.3; gradient, in 16 min from 0% to 95% MPB, flow at 0.9 mL/min. Due to the high salt concentration typically MPA exhibits pH = 12.2 and MPB exhibits pH = 11.7, and they were not adjusted. RNA(2′-OH) 100nt and the truncated 94nt resolve by 0.35 min in a mixture of the two (see under Purification/Fraction Collection using IEX as well as in Figs S5 and S6, Supplementary Information). tRNA *E. Coli* exhibits multiple major peaks by IEX, consistent with composition, and not a major one as observed by IP-RP. mRNA EGFP is composed of one major peak and two minor earlier eluting peaks suggesting multiple components.

It is well known that RNA, even though typically single stranded, exhibits several regions where a part of the strand hybridizes with another part of the strand. A rather striking example is the 3-dimensional structure of tRNA with the shape of a cloverleaf, a shape that is shared by all tRNAs^39^. This shape has functional use in the translational activity of the ribosome, but is not conducive to analysis. In order to detect impurities of closely related materials/oligos, the desired peak as well as the impurities should elute as sharp peaks. This is only feasible, once RNA is “linearized”. Several conditions, either alone or working in sync, lead to linearization, among them the addition of 5 to 6 M urea or 10% formamide. Even though urea and formamide are typically used by molecular biologists, they are disfavored by analytical scientists, most likely due to issues with increasing HPLC column back pressure and peak broadening effects. Parameters exploited often by analytical scientists are: the presence of an organic cosolvent, high temperature, and MP at pH 12. While organic cosolvent, and temperature may work in sync to reduce the stability of self-structured conformers (Figs 2 and S1 (Supplementary Information)), MP at pH 12 alone yields random coil RNA with sharp chromatographic peaks (Fig. 3). Aqueous 10 mM NaOH or 10 mM KOH, measure pH 12.0, exhibit high buffering capacity, and yield practically 100% deprotonation of Guanosine(N1) and Uridine(N3) (both pKas≅10) with concomitant disruption of the G:C and U:A base-pairing^43^. Base stacking is also practically eliminated at pH 12 due to repulsion between the negatively charged bases G and U.

The fact that RNA(2′-OH) 100nt and the truncated 94nt resolve in a mixture by 0.35 min by IEX and not by IP-RP analysis strongly supports the proposition to use IEX for long RNA oligos. Practically speaking, the conditions at which increased temperature and/or a certain concentration of an organic cosolvent will yield complete RNA linearization may not be predictable, or even experimentally easy to determine. Hence high temperature and the presence of a cosolvent may not confidently lead to the best chromatography for a long synthetic RNA oligo. It would be reasonable to simply use IEX MP at pH 12 with suitable HPLC columns, but the stability of the RNA phosphodiester bond in base is a major concern.

### Stability of RNA in base: literature and experiments reported here

The relative instability of RNA compared to DNA is well known. To assess whether or not pH 12 eluent in RNA analysis is a usable medium or not, we address this issue in three ways: First we consulted with the literature, second we measured average internucleotide bond stability with a 32nt RNA oligo in pH 12, and third we conducted on-the HPLC column stability in pH 12 for a number of RNAs, spanning the range of 32nt to 4500nt. The following results clearly support pH 12 chromatography for purity evaluation of RNAs.

Detailed studies concluded that the mechanism of the 3′-5′ internucleotide bond cleavage in RNA is specific base catalyzed by the deprotonated ribosyl 2′OH^44,45^, leading to a cyclic intermediate that gets easily cleaved in base to form a mixture of 2′- and 3′-monophosphates. Li and Breaker^44^ conducted their studies using deoxyoligos with a single RNA linkage in the middle and reported at 23 °C, pH 12 with 10 mM KOH a rate of degradation k = 6 × 10^−5^ min^−1^. Kaukinen *et al*. used 2′OMe modified RNA oligos with a single 2′OH base in the middle^45^, conducted their studies at higher temperatures and reported that stability varies depending on the flanking bases^45^. Accounting for the higher temperature some of the degradation rates reported by Kaukinen *et al*.^45^ agree with Li and Breaker^44^. Using k = 6 × 10^−5^ min^−1^ as a representative degradation rate we calculate that at pH 12 and at 23 °C each RNA linkage will degrade at about 6 × 10^−4^, during the approximate 10 min of HPLC analysis until the RNA of interest elutes from the column. One way to look at this result is to think that an RNA internucleotide bond has a chance of 0.0006 to degrade during analysis, or if an RNA molecule is 10,000nt long, then, on average, it will be cut at 6 random bonds. An obvious way to suppress this degradation is to conduct HPLC analysis at a lower temperature. Hence we implemented 10 °C as the HPLC column compartment temperature, and expect about a factor of 2 less degradation compared to 23 °C.

RNA degradation at pH 12 was revisited using the 32nt RNA oligo with better than 85% purity, which allows for monitoring the actual 32nt RNA peak free from impurities. This oligo was incubated in pH 12.0 with 10 mM NaOH and increasing ionic strength by preparing mock solutions with the HPLC eluents at 20% MPB and 50% MPB (MPB is 1.5 M NaCl in 10 mM NaOH). The samples were incubated at 15 °C in the instrument’s autosampler and analyzed automatically every 20 minutes, using a pH 10 sodium carbonate eluent with 10 °C column temperature. Sodium carbonate buffer at pH 10 is an excellent chromatography eluent for short oligos, like the 32nt RNAs, and exhibits a 100-fold less degradation compared to a pH 12 solution. Hence degradation in a pH 10 chromatography is negligible. The data are included in Table 1, and demonstrate that increasing salt (NaCl) concentration in the solution, increases the degradation rate. Since an RNA sample is subject to a salt gradient during analysis, we estimate a degradation rate in between the two tested systems. From Table 1 average degradation rate of the 32nt oligo from the two salt conditions is 0.0017 per min (Table 1: negative slope {a + b}/2), or 0.0017/31 = 5.5 × 10^−5^ per min per bond. Hence 5.5 × 10^−5^ per min as the average degradation rate of an internucleotide bond at 15 °C in pH 12 and in the presence of 35% MPB or the equivalent of 0.53 M NaCl. This result is in excellent agreement with the reported 6.0 × 10^−5^ per min at 23 °C^44^, and suggests that if one were to conduct this experiment at 10 °C, instead of 15 °C, the observed rate would be somewhat lower than 5.5 × 10^−5^ per min (upper limit). Extrapolation of the two experimental values to zero ionic strength (10 mM NaOH with no salt) provides degradation rate 2.1 × 10^−5^ per min per bond at 15 °C; see later for experimental confirmation of this estimate.

**Table 1:** RNA stability data in pH 12.0, in vials at 15 °C and on-the-column stability at 10 °C.

RNA	Condition	Incubation, min^1^	Total area, HPLC units at 260 nm^2^	Main peak, fraction of total area^2,3^	Notes
32nt Oligo1	20%MPB-15 °C	X	2951	0.7553	a, c
		X + 20	2972	0.7340	
		X + 40	2951	0.7087	
		X + 60	2931	0.6801	
	50%MPB-15 °C	Y	2909	0.8057	b, c
		Y + 20	2896	0.7482	
		Y + 40	2801	0.7231	
		Y + 60	2850	0.6701	
	Regular analysis at 10 °C	—	1797	0.949	d
	Extra 15 min	+15	1893	0.932	
	Extra 30 min	+30	1891	0.929	
RNA(2′OH) 100nt	Regular analysis at 10 °C	—	14420	0.854	e
	Extra 15 min	+15	13937	0.858	
	Extra 30 min	+30	13973	0.852	
mRNA EGFP	Regular analysis at 10 °C	—	4889	0.635	f
	Repeat^4^	—	6684	0.655	
	Extra 15 min	+15	5580	0.600	
	Extra 30 min	+30	5978	0.591	
mRNA Cas9	Regular analysis at 10 °C	—	6103	0.478	g
	Repeat^4^	—	6497	0.472	
	Extra 15 min	+15	5916	0.423	
	Extra 30 min	+30	5812	0.402	

^1^X and Y are a few min worth of incubation at RT before the sample is injected and analyzed.

^2^automatic integration.

^3^Fraction of total area under the main peak, as integrated automatically, does not represent purity.

^4^Comparison between repeat and regular analysis illustrates typical reproducibility and indicates that experimental error is within 1% of the main peak.

^a^Least squares analysis (LSA) of the four points of the main peak with time exhibits degradation rate as the negative slope with slope = −0.00125 per min and R^2^ = 0.996. Degradation rates are determined as percent change which is identical to natural logarithm (ln) change for the small changes observed here.

^b^LSA of the four points of the main peak with time exhibits degradation rate at 0.00216 per min and R^2^ = 0.980.

^c^Linear extrapolation of the two degradation rates from a, b to zero salt provides rate = 0.00065 per min (15 °C).

^d^LSA of the three points of the main peak fraction area with time exhibits degradation rate at = 0.00047 per min (10 °C) in zero salt and R^2^ = 0.993. Please note the excellent agreement between c and d.

^e^Data appear constant within experimental error less than 0.01, likely the result of low resolution of the main peak.

^f^LSA of the main peak fraction with time exhibits apparent degradation rate at 0.0019 per min and R^2^ = 0.838.

^g^LSA of the last peak (not the main peak) with time exhibits apparent degradation rate at 0.0025 per min and R^2^ = 0.953, faster than the one observed with mRNA EGFP.

We also designed a protocol to test degradation of any RNA at pH 12.0 in 10 mM NaOH, and assess directly extent of the on-the-column degradation; this protocol could be used with any IEX column qualified for pH 12 MP. The on-the-column stability method involves delaying the onset of the salt gradient, while keeping the gradient steepness the same, and results, in the absence of degradation, to an identical chromatographic profile, but shifted in time. Any detectable degradation due to additional pH 12 exposure, will appear as a decrease of the main peak and as an increase in degradants, i.e. small peaks eluting ahead of the main peak. The advantage of running the on-the-column stability is that it can be done automatically for any RNA sample. A visual comparison of the main peak height and the absence of degradants in the HPLC region in the 3 to 4 minutes elution area ahead of the main peak provide unequivocal evidence for the usability of the IEX pH 12 method. On-the-column stability can be conducted only in the absence of salt. Using a buffered eluent, even at concentrations as low as 20 mM buffer, broadens the HPLC peak and alters slightly the profile, due to the added salt that the sample is exposed to, and therefore prevents quantitative comparison.

**Figure 4:**
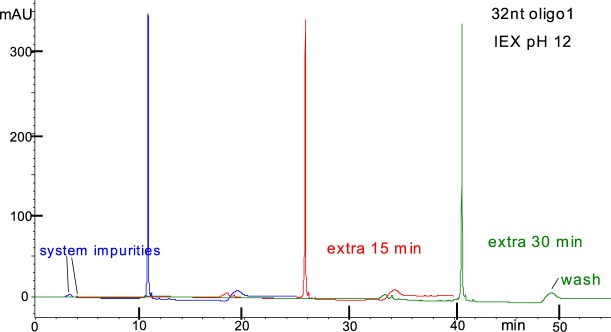
DNAPacIEX on-the-column stability of a 32nt RNA oligo1 in pH 12 at 10 °C; a slight decrease of the main peak is detectable (quantitation of fraction peak area in Table 1). Same sample used for three consecutive runs with the same instrument and MP. Blue trace, same method as in Fig. 3. Red trace, method as in Fig. 3, but with delay of the gradient by 15 min isocratic at 0% MPB. Green trace, method as in Fig. 3, but with delay of the gradient by 30 min isocratic at 0% MPB. Methods end with a 1 minute wash (see small hump) at the high percent MPB followed by 7 min equilibration of the column to initial conditions, i.e. 0% MPB. System impurities, elute at about 3.5 min, and are attributed to the water source; these impurities do not show up when the initial conditions include low salt concentration.

Figure 4 illustrates “on-the-column” stability experiment for the 32nt RNA oligo for an extra 15 min and an extra 30 min in 10 mM NaOH at 10 °C. A small decrease of the main peak with time is visible and confirmed by quantitative integration of the peaks (see Table 1). The decrease of the main peak with time, even though it is within experimental error of about 1%, is consistent with degradation with a rate of 0.00047 per min for the 32nt oligo, or the equivalent of 0.00047/31 = 1.5 × 10^−5^ per min (lower limit) per bond at 10 °C. Please note that the two experimentally obtained degradation rates in the absence of salt are in good agreement, i.e. 0.00047 per min at 10 °C (d in Table 1) and 0.00065 per min at 15 °C (c in Table 1).

### sgRNA analysis

RNA(2′OH), 100nt and 94nt, and 100nt RNA(2′OMe) (see Materials and Methods) were analyzed by both IP-RP and IEX methods. The HPLC profiles in neutral pH (Fig. 1) led us to pursue analysis in basic pH. Considering that sgRNA is currently promoted as pharmaceutical, suitable HPLC methods for analysis and purity determination are sought for. Inexperienced analysts may think that one can pick an HPLC column and by changing parameters, such as buffer concentration and pH, one may develop a good analytical method; reality couldn’t be any further. One should select an HPLC column/mode, based on literature, application notes, and earlier experience. The actual method development will follow once the HPLC column has been qualified for the specific application. Here we illustrated this approach (see Figs 2 and 3 and text), using a general method with a typical MP for each column, and a relatively steep gradient so that all RNA materials elute quantitatively from the column, as shown by blank runs in between sample analyses. A comparable effort could be done with two HPLC columns of the same mode, in order to select one or the other. Once the mode and the specific column are established, method development follows. For example, IEX parameters to optimize for sgRNA analysis are: salt (typically NaCl or NaClO_4_), choice of buffer, the presence/absence of a cosolvent, initial MP composition, and gradient steepness. HPLC methods for purity analysis should run about 30 min or less, including equilibration to initial conditions.

**Figure 5:**
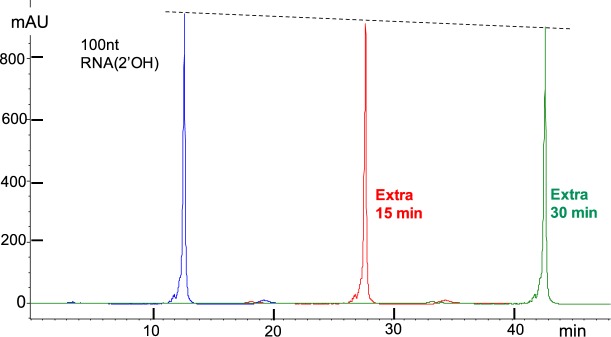
DNAPacIEX HPLC profiles and on-the-column stability of 100nt RNA(2′OH) in pH 12 at 10 °C; see also Fig. S3 (Supplementary Information). Quantitation in Table 1 shows no trend, but the dashed straight line of the peak height indicates a slight decrease with time, consistent with on-the-column degradation. Analytical conditions identical to the ones described in the captions of Figs 3 and 4.

On-the-column stability was tested with 100nt RNA(2′OH) (Figs 5 and S3) and with RNA(2′OMe) (Fig. S4) (see Supplementary Information) at 10 °C with the same analytical conditions, as described in the captions of Figs 3 and 4. The results are barely outside experimental variation but the trend is presumed to be due to degradation. RNA(2′OH) is expected, and found, to degrade more than RNA(2′OMe), because 2′-OMe can’t get deprotonated and/or act as a nucleophile^44^. An important question for RNA analysis in a pH 12 medium is what are the majority of the degradants and whether or not the presence of the degradants alters the HPLC profile of the sample. One might think that the longer the RNA, the more degradants, in the form of shorter lengths, will accumulate. However this is not borne out by our studies, as described below under “mRNA quality evaluation”.

### Purification/Fraction Collection using IEX

It is desirable to scale up the best analytical method and use it to do fraction collection in an effort to obtain purer RNA, and this is even more important now for sgRNAs targeting FDA approval. Because we do not have a fraction collection module on our HPLC instrument, we did it manually collecting approximately 0.25 mL in a preweighted test tube right after the detector. The exact weight of the fraction was obtained and the corresponding HCl to neutralize it was added, followed by addition of TRIS.HCl buffer to yield an approximate 25 mM pH 8 buffered solution that was then subject to salt removal by TrimGen purification (See Materials and Methods). The process lasted no more than 20 min per sample, and was implemented with both the 100nt RNA(2′OH) and the truncated 94nt RNA(2′OH). Both materials were obtained in good purity as seen in Figs S5 and S6 (Supplementary Information). Our process could be further improved by automation and preparative IEX HPLC at pH 12 could be used for sgRNA purification.

### tRNA analysis

Analysis of tRNA *E. Coli*, a mixture of over 40 components, was conducted by both IP-RP (Fig. 2) and IEX (Fig. 3) using different methods, but comparable from the point of view of percent change of the gradient. Visual comparison of the two figures illustrates that IEX exhibits superior resolution compared to IP-RP. Further method development can be done with either chromatography, but method development may not change the superiority of IEX. On the column stability testing of a tRNA *E. Coli* sample at pH 12, with the same protocol, as used earlier for the 32nt and the 100nt RNAs, is depicted in Fig. S7 (Supplementary Information) and no detectable degradation was observed. For this material we optimized the analytical conditions and obtained an HPLC profile with over 40 distinct, but not baseline resolvable, peaks consistent with known composition of tRNA E. Coli (see Fig. S8, Supplementary Information). Automated fraction collection using the above protocol and a preparative column may yield a number of relatively pure individual tRNAs.

**Figure 6:**
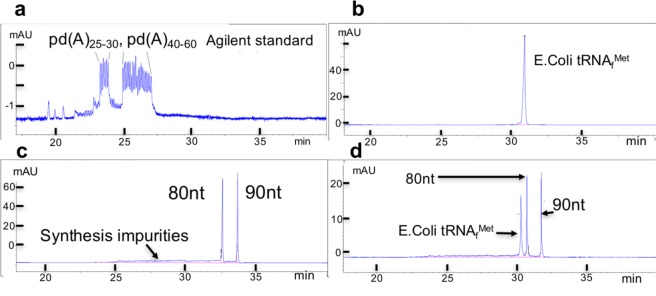
CGE analyses of standard, deoxy oligos, and purified tRNA_f_^Met^ (**a**) CGE analysis of standard to show that peaks of oligodeoxyadenylates, standard supplied by Agilent Technologies, exhibit migration times (mt) that differ by 0.1 min between N and N + 1, independent of length within the range of the standard. (**b**) *E. Coli* tRNA_f_^Met^ exhibits a single peak consistent with high purity. (**c**) Deoxyoligos 80nt and 90nt, of different sequences, single peaks with tails migrating earlier than the main peak consistent with impurities derived from incomplete synthesis; the mts of the two oligos differ by 1.0 min consistent with their 10nt difference in length. (**d**) Sample is mixture of the two deoxyoligos and the tRNA. tRNA_f_^Met^ migrates 0.4 min ahead of the 80nt oligo, consistent with a length of 76nt. These experiments suggest that the specific CGE method resolves oligos based strictly on length with constant resolution of 0.1 min between N and N + 1 for the range of 25 < N < 90, and independent of sequence, or of linkage (ribo vs deoxyribo). CGE method using μPAGE-3 product and standard supplied by Agilent (see Materials and Methods). Product has been discontinued and was not available during the current study in order to test representative sgRNAs.

Excellent resolution with up to 60nt long RNA oligos was reported recently using CGE with a polyvinylalcohol coated fused silica capillary^33^. It is likely that this method could be extended to include longer RNAs, and yield a strictly size-dependent separation, well documented for CGE. In our own Laboratory both length and purity determination was accomplished for a relatively pure sample of tRNA_f_^Met^ using all DNA standards and a ready coated CGE capillary (Fig. 6), a product that has been discontinued. CGE analysis with commercially available ready-coated capillaries could provide the best analysis of sgRNAs.

### mRNA quality analysis

**Figure 7:**
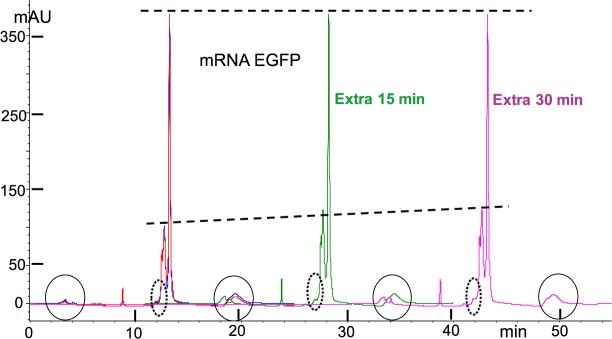
DNAPacIEX on-the-column stability of 996nt mRNA EGFP, in pH 12 MP at 10 °C. mRNA EGFP elutes as three peaks, a main peak and two smaller ones eluting ahead of the main peak and this profile remains unchanged with prolonged analysis. Method as described in captions of Figs 3 and 4. Small bumps in circles at the baseline are system impurities and method wash discussed in earlier captions. Blue and red trace demonstrate the method’s reproducibility. HPLC profiles were artificially brought to the same highest peak level (top dotted line) in order to minimize sample variability and focus all the change into the minor components (see lower dotted line with positive slope). Quantitation of the changes as fraction of area of main peak in Table 1; the height of the minor peaks is practically a better estimate compared to peak area (see Table 1). No new/increasing peaks, i.e. degradants, were observed as a function of time in the HPLC region ahead of the peaks (dotted ovals), even though mRNA process impurities exist in that region, but do not increase with time.

**Figure 8:**
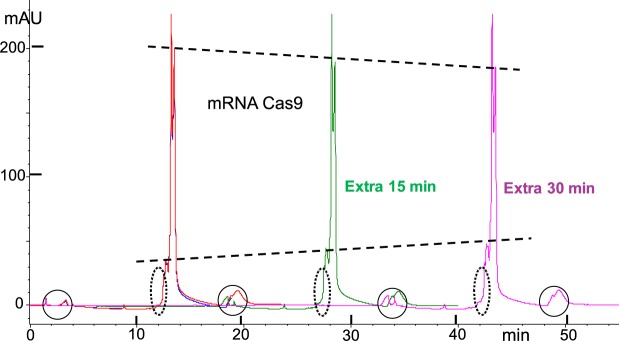
DNAPacIEX on-the-column stability of 4,500nt mRNA Cas9, in pH 12 MP at 10 °C. mRNA Cas9 elutes as three peaks, two major peaks and a smaller one eluting first; this profile remains unchanged with prolonged analysis. Methods as described in captions of Figs 3 and 4. Overlapping HPLC profiles illustrate the method’s reproducibility (red and blue traces). Peaks in circles identified as system impurity and wash were discussed in the caption of Fig. 4. HPLC profiles were artificially brought to the same highest peak level (middle peak) in order to minimize sample variability and focus all the changes into the other two components (see dotted line with negative slope attributed to longer material degradation, and dotted line with positive slope for shorter material accumulation). Quantitation of the changes in Table 1. No peaks are detected in the HPLC region (dotted ovals) ahead of the early eluting component, where degradants are expected.

Exploiting the DNAPacIEX method with up to 4,500nt mRNAs resulted in reproducible analysis, and quantitative elution of mRNAs from the column. We analyzed a number of mRNA Cas9 batches obtained from a collaborator and found that the column/method discriminates monomers, short sequences and other small molecules, resulting from transcription and incomplete purification. Whether or not the manufactured mRNA is devoid of process intermediates is a valuable piece of information, and hence the above IEX method (see caption of Fig. 3) can be exploited for quality control. The simple fact that we obtained analyses of mRNA Cas9 batches that did not show minor peaks ahead of the main peaks, even under the highest sample load of about 80 μg, is consistent with the relative stability of mRNAs in a pH 12 MP (Figs 7 and 8). Resolution of shorter mRNAs in the 1000 nt range appears to be better, as shown by the three practically fully resolvable peaks with the mRNA EGFP sample (Fig. 7). Even the HPLC profile using DNAPacRP exhibits a hump in front of the main peak of mRNA EGFP (Fig. 2), and illustrates agreement between the two orthogonal chromatography modes. Figures 7 and 8 illustrate the excellent reproducibility of mRNA IEX HPLC profiles (see overlapping blue and red traces). Table 1 lists fraction of HPLC area at 260 nm for the last eluting peak and provides evidence for material shift from the last peak to the earlier eluting one(s). No degradation was detected in the HPLC region where shorter sequences would elute, even though material shift from the presumably longer material to the shorter material is evident with both mRNAs.

Why is degradation and/or shift of material sometimes detectable and sometimes not? There are a number of parameters critical for degradation and its detection: These parameters are (i) the degradation rate under the specific analytical conditions, (ii) the length of the RNA, (iii) the sample load, and (iv) the resolution of the HPLC column for the tested material. In addition there is a statistical factor that will be discussed later. Both literature values and our own experiments suggest an upper limit degradation rate of 5.5 × 10^−5^ per min per bond at 10 °C in the presence of 0.52 M NaCl (≈35% MPB). For a typical 10 min analysis time, this rate translates to 5.5 × 10^−4^ degradation per bond, which is negligible degradation for any oligo. It could be a measurable quantity in the case of a pure oligo, a resolving HPLC column, and a high sample load. For example, a pure 100nt oligo would exhibit a single bond cleavage in only 5.5% of the molecules which is barely outside experimental error. Moreover if the 100nt oligo is composed of, let us say, 50% 2′-OMe substitutions, then degradation should measure only 2.75%. Such low level of degradation in pH 12 may be considered negligible, and the HPLC method scaled up and used for isolation/purification, as long as fraction collection is conducted using a suitably neutralizing and buffered solution for collection. If IEX HPLC indicates that the specific sgRNA is not of acceptable purity, and if manufacturing process development does not lead to improvements, then synthesis of two shorter fragments and chemical ligation^46,47^ may be a better solution to obtain quality material.

Degradation becomes detectable with a 1000nt long RNA, such as the mRNA EGFP, primarily because 55% of the molecules will exhibit on average a single bond cleavage. Consequently, if the molecule was a pure 1000nt long RNA and if the HPLC method could resolve a 999nt long from a 1000nt long molecule, then degradation would be measurable. The reality, as it is seen in Fig. 7, is that man-made (here transcribed) RNAs are typically composed of different lengths and degradation becomes visible as a shift of the material from longer to shorter ones. A similar pattern of shifting material, is observed with mRNA Cas9 (Fig. 8), and for this 4,500 long RNA the degradation rate predicts that every molecule will be subject on average to 2.5 bond cleavages. Inspection of Fig. 8 indicates that the HPLC profile is barely changing and that no short, oligo-type, degradants are detectable. One way to reconcile “severe” degradation and minimal detection of it, is to consider it from a statistical point of view, namely a 4,500nt long molecule will produce a large number of long cuts and a disproportionally small number of short cuts. The longer the molecule, the fewer the small cuts, simply because small cuts come predominantly out of the two ends and that contribution becomes increasingly less as the length of the polymer increases. Long cuts are proportionally more detectable than short cuts because absorbance is proportional to the number of monomer/nucleotides, and thus short cuts are doubly compromised. In summary, the ability of 10 mM NaOH (pH 12) to linearize RNA, and the slow degradation of RNA in such medium, in combination with the limited resolution of HPLC IEX packings for long RNAs, results in reproducible chromatography that depicts the broad distribution of an RNA sample and may serve as a quality control method and/or a batch-to-batch comparison.

## Conclusions

Well resolving HPLC methods must be developed and implemented to facilitate process improvements in synthetic or transcription protocols of man-made RNAs. IEX chromatography at pH 12 was extensively used for RNA analysis during the period of 1970 to 1990, and the current study confirms its usability and superiority compared to IP-RP. Our study provided an experimental approach of how-to select chromatography mode and HPLC column for a specific application. It also established that the most efficacious denaturing medium for HPLC analysis of RNA is aqueous pH 12 at 10 °C, and showcased a protocol for testing on-the-column stability with any RNA material, with no special requirement for the IEX HPLC column, besides its compatibility with pH 12. We measured an upper limit of 5.5 × 10^−5^ per min for RNA internucleotide bond degradation in 10 mM NaOH at 15 °C in the presence of 0.5 M NaCl, and a lower limit of 1.5 × 10^−5^ per min per bond in 10 mM NaOH at 10 °C. These values translate to practically negligible degradation of an RNA oligo during HPLC analysis time. With long RNAs, such as mRNAs, degradation appears as a small shift of the material from longer to shorter lengths, but does not alter the overall HPLC profile and does not produce detectable short oligo degradants. This experimentally shown phenomenon is consistent with the statistical notion that the longer the RNA, the fewer the short sequences produced by degradation. The relative stability of RNA oligos in basic IEX HPLC can be exploited for purity/impurity analysis, batch-to-batch comparison, as well as for fraction collection as long as care has been taken that the isolated fractions are stored in a neutral medium. With mRNAs pH 12 IEX HPLC is a suitable method for identifying low molecular weight process development contaminants, as well as for quality control as shown by the reproducibility of the HPLC profile. Since RNAs are moving in the realm of pharmaceuticals, high purity materials are required for testing in *in-vitro* and *in-vivo* assays and in order to minimize the sources for off-target effects^48^. Purity analysis of such RNAs should be conducted using HPLC methods with documented resolving power.

## Materials and Methods

Custom-made RNA 32nt oligos and mRNAs (EGFP CleanCap™ and Cas9 CleanCap™) were purchased from TriLink Biotechnologies. Oligo purity was determined by us. Oligo1, sequence: AGA GAG CCC CAG AGA GCC CCA GAG AGC CUU CA. Oligo2, sequence: AGA GAG **AG**C CAG AGA G**AG** CCA GAG AGC CUU CA bold indicates the four different bases, purines in Oligo2 replacing four Cs in Oligo1.

Custom-made 100nt and 94nt Oligos were purchased from Dharmacon. RNA(2′OH) 100nt sequence: 5′-UUA CAG CCA CGU CUA CAG CAG UUU UAG AGC UAG AAA UAG CAA GUU AAA AUA AGG CUA GUC CGU UAU CAA CUU GAA AAA GUG GCA CCG AGU CGG UGC UUU U-3′. RNA(2′OH) 94nt sequence: same as RNA(2′OH) 100nt but missing the 6 nucleotides at the 3′end. Please note that out of the six, five are pyrimidines and found by us to be important for secondary structure (S1, Supplementary Information and relevant text). RNA(2′OMe) 100nt sequence: 5′-mUmUmA CAG CCA CGU CUA CAG CAG UUU UAG AmGmC mUmAmG mAmAmA mUmAmG mCAA GUU AAA AUA AGG CUA GUC CGU UAU CAmA mCmUmU mGmAmA mAmAmA mGmUmG mGmCmA mCmCmG mAmGmU mCmGmG mUmGmC mUmUmU mU-3′; m stands for 2′OMe. 2′OMe is used frequently in sgRNAs in order to suppress *in vivo* degradation and this is why we studied it here.

RNAs were diluted with Ambion Nuclease-free water, not DEPC treated, from Thermo Fisher Scientific to 200 μM stock solutions and stored at −20 °C. tRNA *E. Coli* was purchased from Sigma and tRNA_f_^Met^ *E. Coli* was a gift from Professor Harry Noller of the University of California in Santa Cruz, and shared with us by Dr. Christopher Murray of Galen Biotechnologies.

Triethylamine acetate (TEAA) buffer 2 M was purchased from Life Technologies. TRIS.HCl 1.0 M buffer pH 7.0 was purchased from Sigma and same buffer pH 8.0 Ultrapure from Invitrogen. NaCl crystal ACS min 99.0%, and sodium perchlorate monohydrate ACS, 85 to 90%, from Alfa Aesar. Sodium hydroxide solution and Hydrochloric acid solution both at 1.0 N, both BioReagent purchased from Sigma. Sodium phosphate dibasic anhydrous AR granular purchased from Mallinckrodt (phosphate buffer at pH 12 was found to degrade RNA, even at very low buffer concentrations; the phenomenon was not retested with phosphate buffer of another grade). Sodium carbonate and sodium bicarbonate, both BioXtra, from Sigma Aldrich. Distilled water from ArrowHead or Alhambra was used for preparation of HPLC MP.

Spin columns (TC-100 FC from TrimGen Corporation) were used to remove salt and buffer according to the manufacturer’s instructions; practically 100% recovery of RNA is achieved with minor volume/concentration changes.

pH measurements were conducted with an Orion 3 pH meter equipped with a Gel pH electrode 9146BN from Thermo Scientific after 3-point calibration with standards LSS purchased from Grainger.

As the primary analytical tool, we used an Agilent 1100/1200 LC HPLC equipped with a binary pump, Diode Array Detector (DAD), a 1290 Infinity Autosampler/Thermostat, and Chemstation software Rev.B.04.01 SP1 for data acquisition and processing. Both the autosampler and the column compartment have individual temperature control. Typically the autosampler was kept at 15 °C, unless otherwise noted.

HPLC columns, DNAPac PA200 from ThermoFisher Scientific (Dionex), were used with configurations 2 × 250 mm or 4 × 250 mm, typically at about 0.5 mL or 0.9 mL flow per min, respectively. A second HPLC column used was from the same supplier, DNAPac RP in the configuration of 2.1 × 100 mm and used with the flow of about 0.35 mL. The first type of column is used for IEX and the second for IP-RP chromatography. The performance of the instrument and the column was qualified using standards every time ahead as well as after analysis of samples. Mobile phase was prepared and the pH was measured. The same buffer was contained in both MPA and MPB. TEAA buffer was used for IP-RP, typically at 100 mM concentration. As buffers for IEX we used TRIS.HCl at pH 7.0 and at pH 8.0, sodium carbonate at pH 10.0 and at pH 11.0, sodium phosphate at pH 12.0; all these buffers were used at 25 mM concentration in both MPA and MPB. 10 mM NaOH was used as a pH 12.0 eluent; no buffer necessary. The pH values were acceptable in the range pH ± 0.3, and typically not adjusted. The HPLC methods reported here can be used for RNA of any length, and are not optimized for individual RNAs, unless noted otherwise.

Selected analyses were conducted with an Agilent G1600 Capillary Electrophoresis (CE) instrument equipped with DAD and Chemstation software Rev.B.04.03 for data acquisition and processing; the CE was used in conjunction with a circulating bath to control the autosampler’s temperature, typically kept at 15 °C. The capillary’s temperature was controlled by the instrument’s software. Typical capillary zone electrophoresis (CZE) analyses were conducted with an untreated fused silica capillary (50 μm × 40 cm) with an extended light path purchased from Agilent Technologies in pH 9.3 50 mM sodium tetraborate buffer as detailed elsewhere^42^. CGE method was conducted with product μPAGE-3 (PN 191–3211), a 75 μx75 cm capillary preloaded with 3%T, 3%C polyacrylamide gel and with buffer, TRIS-borate/urea pH 8.3 and poly(A) standard supplied by Agilent and following supplier’s protocol for analysis.

Both CE and HPLC peaks were detected and identified using the DAD in the UV–vis region 200–450 nm and the electropherograms and chromatograms were recorded among other wavelengths at 260 nm; the latter are reported here.

## Supplementary information

HPLC methods for purity evaluation of man-made single-stranded RNAs

## Data Availability

All data generated during this study are included in this published article (and its Supplementary Information files). Additional information can be obtained directly from the author upon request.
