# Supplementary material for: HPLC methods for purity evaluation of man-made single-stranded RNAs
Source: Sci Rep. 2019 Jan 31;9:1019. doi: 10.1038/s41598-018-37642-z (PMC6356003; doi:10.1038/s41598-018-37642-z)
Supplement: Supplementary file 1 — HPLC methods for purity evaluation of man-made single-stranded RNAs [file 41598_2018_37642_MOESM1_ESM.pdf]

Supplementary Information

HPLC methods for purity evaluation of man-made single-stranded RNAs

Anastassia Kanavarioti
Yenos Analytical LLC, 4659 Golden Foothill Pkwy, Suite 101, El Dorado Hills, CA 95762, USA
Correspondence should be addressed to A.K. (email: tessi.kanavarioti@gmail.com)

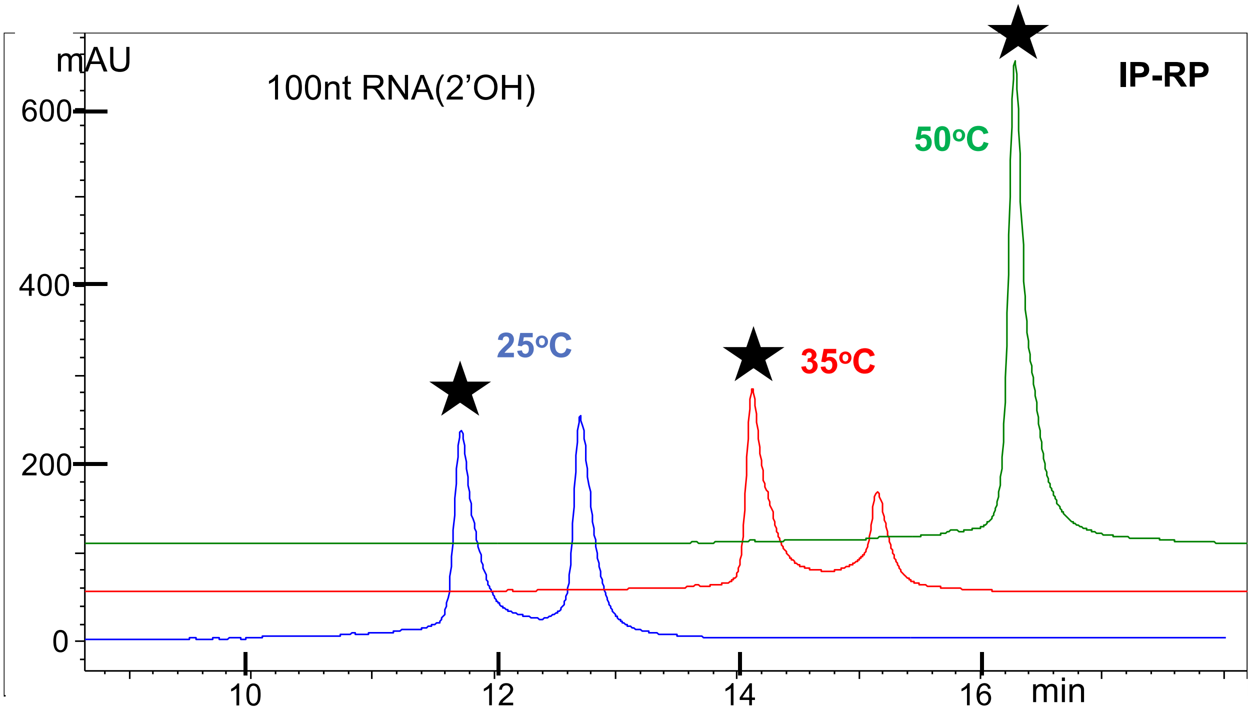

Figure S1: HPLC IP-RP profile of 100nt RNA(2'OH) exhibits two peaks, and with increasing temperature material shifts quantitatively from the late to the early peak. Comparable profiles are exhibited by the 100nt RNA(2'OMe), but not by the 94nt RNA(2'OH) which appears as a single, relatively broad peak, independent of temperature. These observations are consistent with the presence of, at least two, stable conformers. Chromatography conducted using DNAPacRP column; flow at 0.30mL/min, MPA 0.1M TEAA, MPB 25-75% CH_3CN-H_2O (v/v) in MPA; gradient, 15% to 75% MPB in 16 min. Retention times (r.t) in min correspond to the analysis at 25°C. Profiles at the higher temperatures are shifted for clarity; in reality peaks practically overlap. At 50°C all the material elutes with the r.t. of the early peak (see star). The presence of two stable conformers in both 100nt RNAs, the canonical and the heavily methylated, but not in the 94nt canonical RNA suggests that the 6 nucleotides at the 3'end of the sequence are critical for secondary structure.

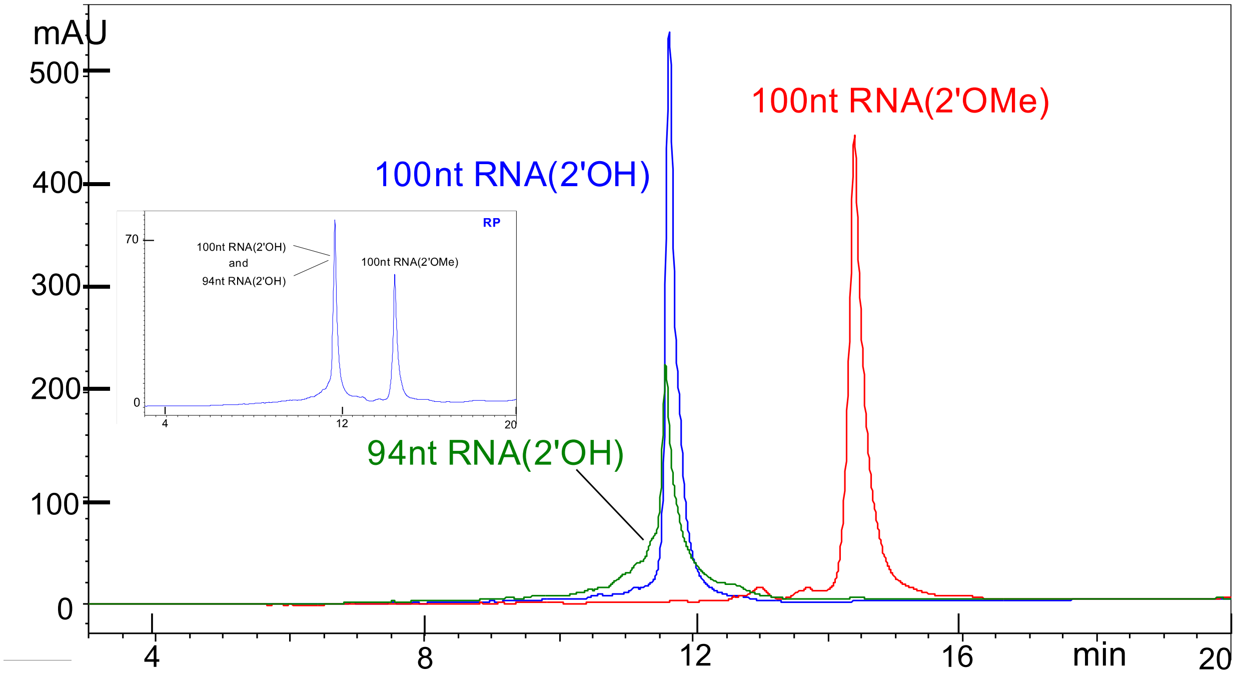

Figure S2: DNAPacRP IP-RP HPLC profiles of the three sgRNAs analyzed with the same method at 50°C with CH_3CN as cosolvent. MPA, 0.1 M TEAA pH 7.0 in water; MPB, 25-75% CH_3CN-H_2O (v/v) in MPA; gradient 15% to 75% MPB in 16 min, flow at 0.30 mL/min. Gradient is here shallower compared to the one used for the chromatography in Fig. 2 in an attempt to improve resolution among RNAs of comparable length. As seen in this figure by RP 100nt RNA(2'OH) elutes earlier compared to 100nt RNA(2'OMe), but 100nt RNA(2'OH) and 94nt RNA(2'OH) coelute as shown by the identical r.t. when analyzed separately, and by analysis in a mixture of the three with an approximate 1:1:1 composition and further dilution in water in order to reduce sample concentration and make any tentative separation detectable (see **insert**, y-axis max at 70 mAU).

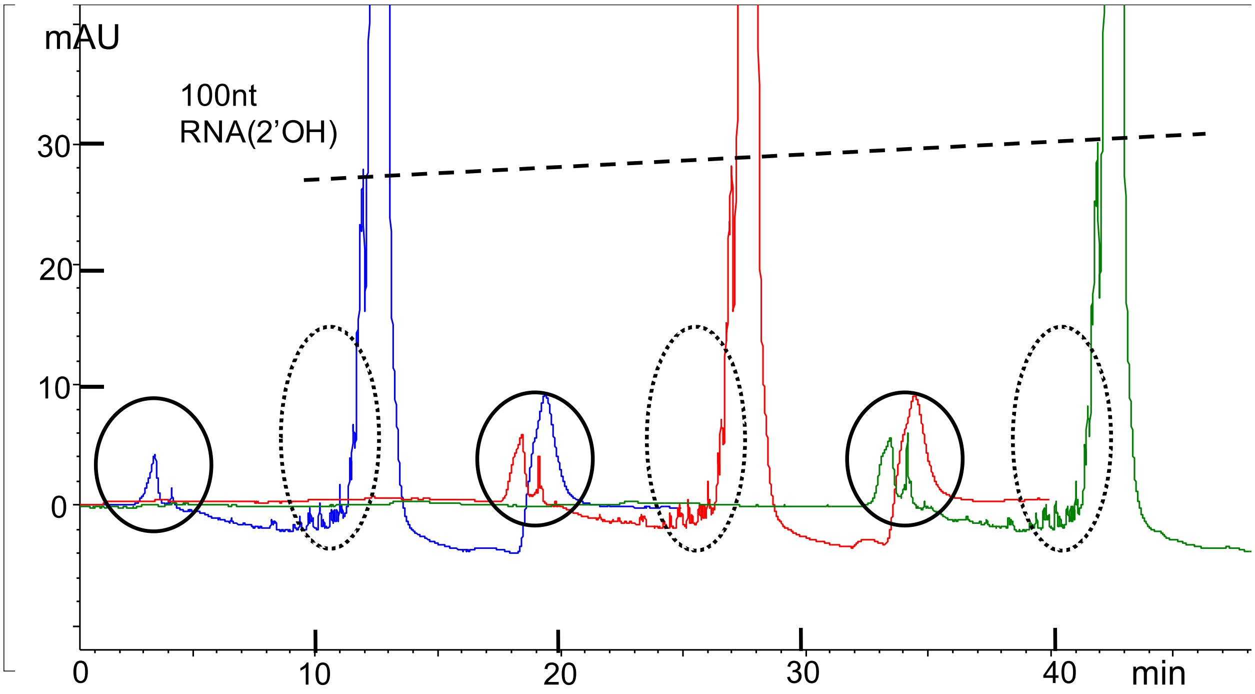

Figure S3: DNAPacIEX on-the-column stability of 100nt RNA(2'OH) in pH 12 MP at 10°C. Magnification of the baseline from Fig. 5 to show no detectable degradation. The small peaks, impurities of the material, eluting ahead of the main components, illustrate the excellent resolution of the HPLC column. However, no new peaks, i.e. degradants as a function of time in pH 12, can be detected ahead of the main components (dotted ovals). System impurities and the "wash" of the column are in solid circles, same for all three analyses (see explanation in Fig.4). The dashed straight line indicates a slight increase of the minor peak, eluting ahead of the main peak, consistent with the observation of a slight decrease in the main peak (see Fig. 5). These trends suggest a shift in material (consistent with degradation) from longer to shorter sequences as a function of the extra time of the material residing on the column in pH 12.

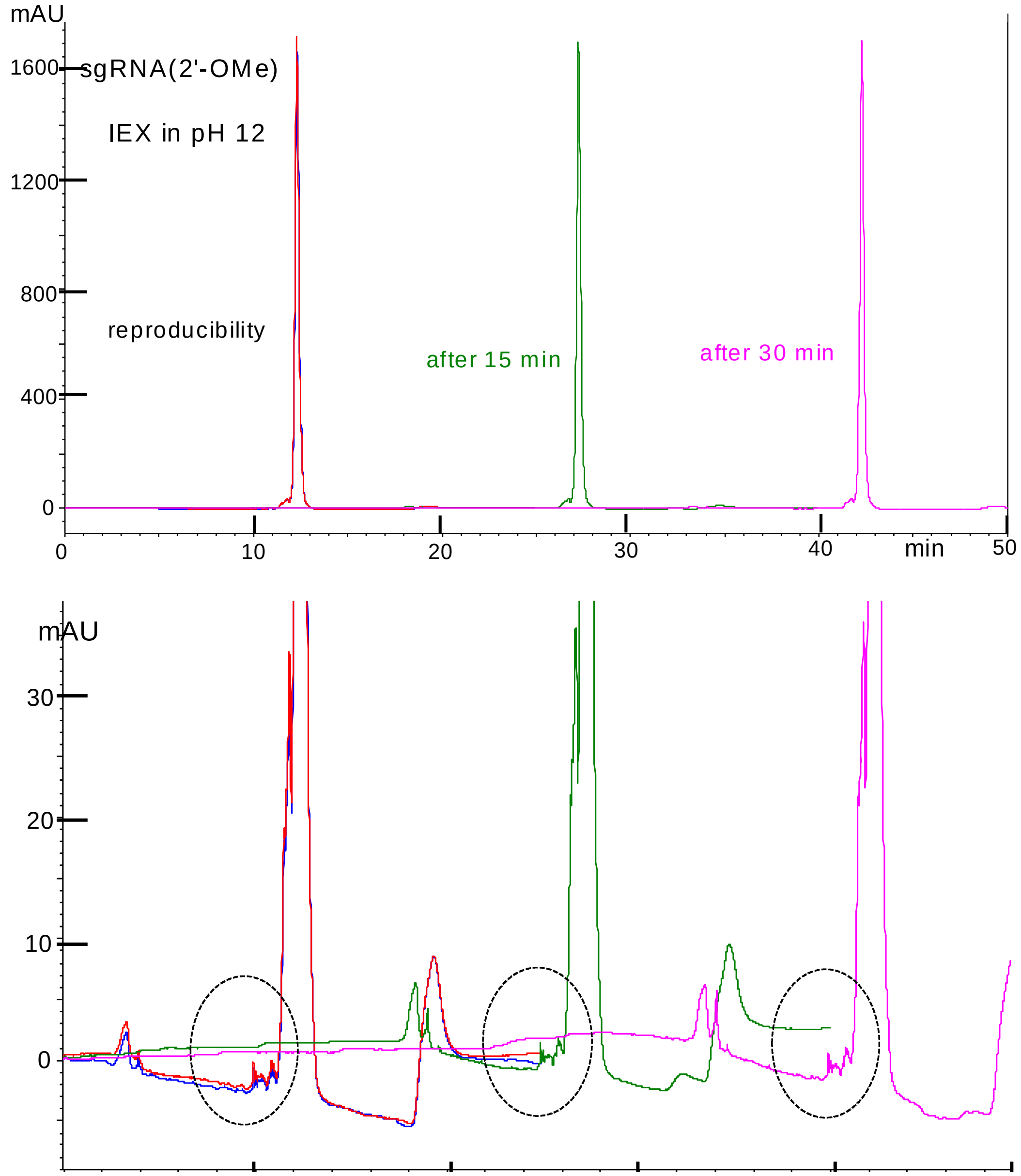

Figure S4: DNAPacIEX on-the-column stability of 100nt RNA(2'-OMe) in pH 12 MP at 10°C. Degradation with RNA(2'OMe) is not observed as seen by the absence of trends in the main peak and in the minor peak confirming the better stability of RNA(2'OMe) compared to RNA(2'OH). HPLC methods as described in captions of Figs 3 and 4. **Top, full HPLC profile:** Same sample analyzed twice with method of Fig. 3 (overlapping blue and red traces) shows the excellent reproducibility of the method. System impurities and method wash, just as seen in Fig. 4, exist, and appear smaller due to the high sample load (see y-axis). Sample load was purposely high to facilitate detection of any changes, but no detectable changes were observed. The absence of detectable degradation is partially attributed to the high percentage of 2'-OMe groups and to the relative broadness of the peak, compared to the peak of the 32nt RNA oligo1. A broad peak for an oligo in pH 12 medium clearly indicates less than optimal purity. **Bottom, magnification of the baseline.** Dotted circles depict the areas where tentative degradants would appear as new or increasing peaks as a function of on-the-column residence time.

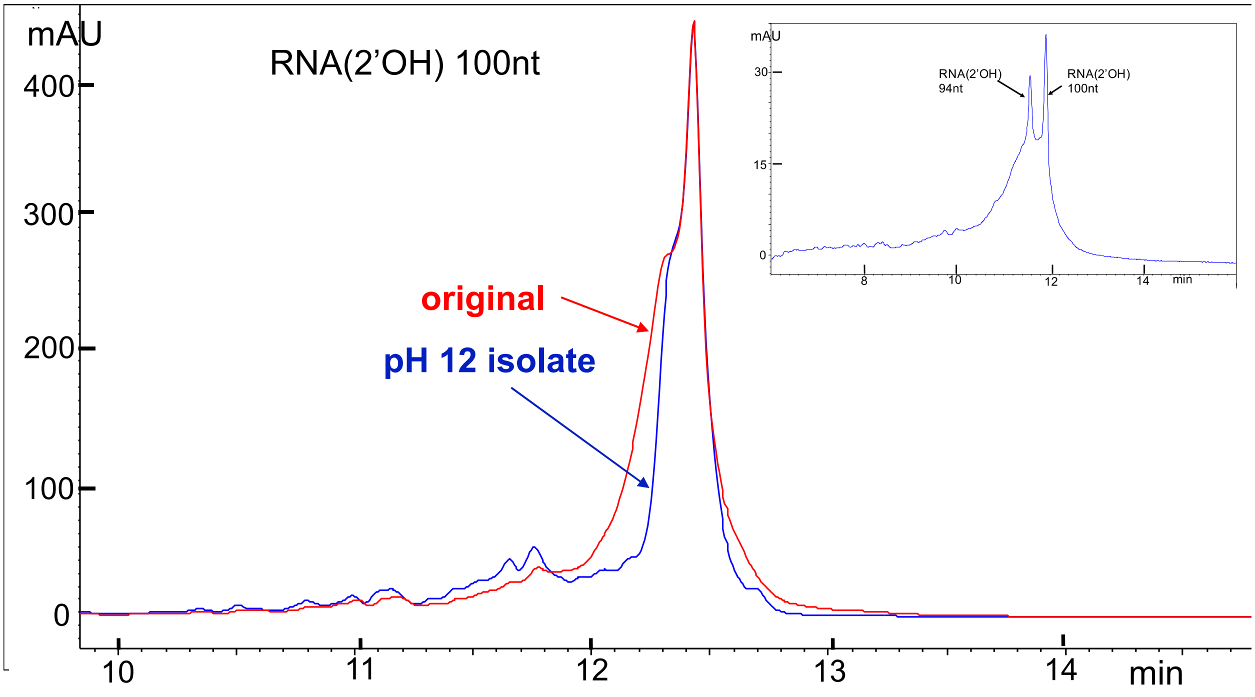

Figure S5: Manual fraction collection to obtain purer 100nt RNA(2'OH) using DNAPacIEX. Red trace original material from supplier, blue trace after isolation at pH 12, neutralization with 0.1 M HCl, addition of a few µL of TRIS.HCl buffer pH 8 to obtain an approximate 25mM buffer, desalting using TrimGen (see Materials and Methods). Analysis of original material at 2 µL, analysis of desalted fraction at 75 µL. Analytical conditions for all analyses/fraction collection: flow at 0.9mL/min, column compartment at 10°C, gradient in 16 min from 35% to 70% of 1.5M NaCl in 0.01 M NaOH, pH 12. **Insert** illustrates the resolution between 100nt RNA(2'OH) from the truncated 94nt RNA(2'OH) by 0.35min (peak to peak) of the original materials in a mixture of the two.

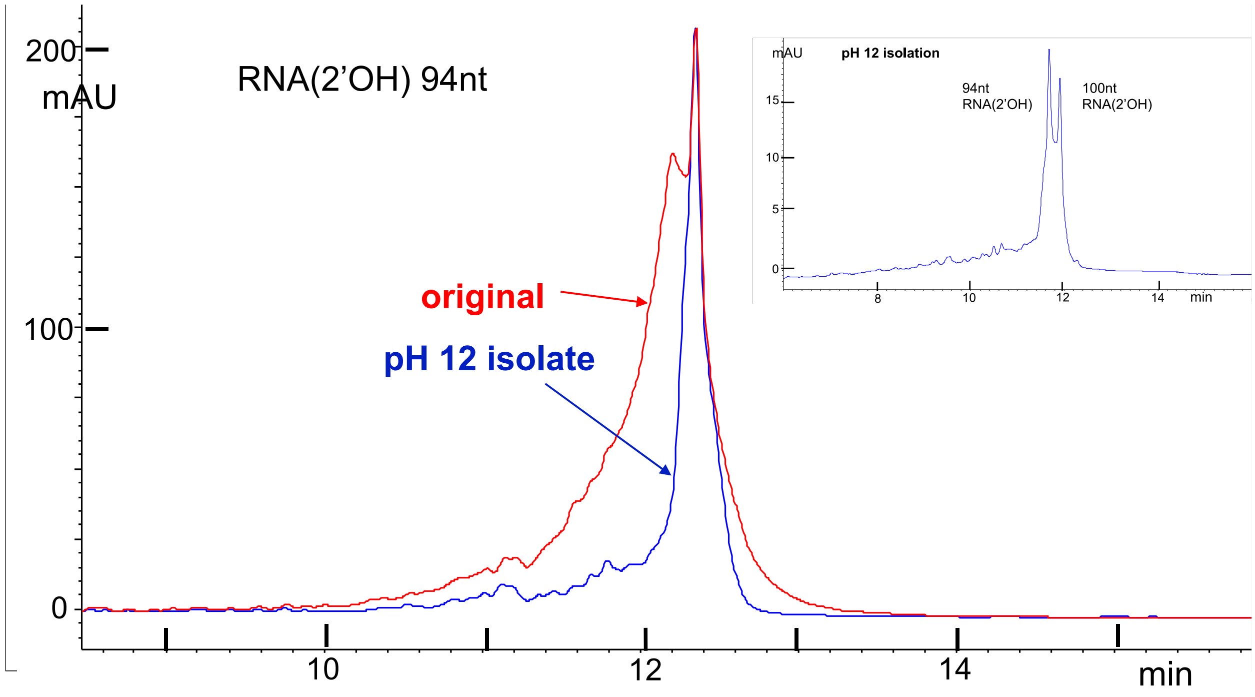

Figure S6: **Manual fraction collection to obtain purer 94nt RNA(2’OH) using DNAPacIEX.** Red trace original material from supplier, blue trace after isolation at pH 12. Analytical conditions and protocol for isolation/desalting identical to the ones described in Fig. S5. **Insert** illustrates the resolution between the 100nt RNA(2’OH) from the truncated 94nt RNA(2’OH) of the manually collected fractions after desalting in a mixture of the two. Analytical condition as in Fig S5, but flow at 0.8mL/min.

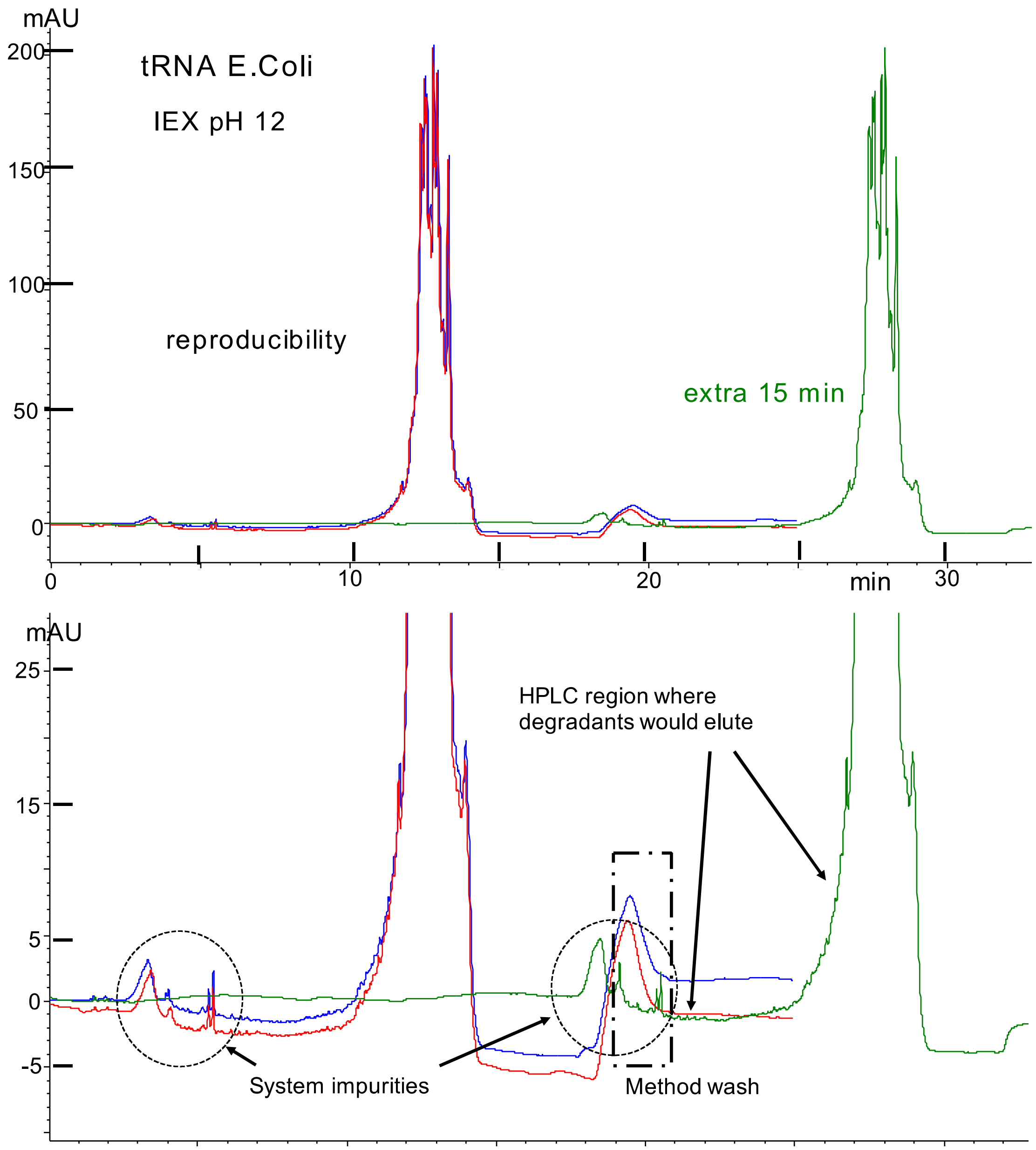

Figure S7: DNAPacIEX on-the-column stability of tRNA E.Coli in a pH 12 MP at 10°C. Top, full HPLC profile; Bottom, magnification of the baseline. HPLC methods as described in captions of Figs 3 and 4. Even though tRNA E.Coli is a mixture of over 40 tRNAs, about 76nt long, with potential degradants easily resolvable by this chromatography, no degradants were observed after 15 min extra analysis time in 10mM NaOH. Same sample analyzed twice (overlapping blue and red traces) shows the excellent reproducibility of the method. Peaks that correspond to system impurities in dotted circles and method wash in rectangle, do not interfere with the region where tentative degradants would elute, i.e. area between the arrows.

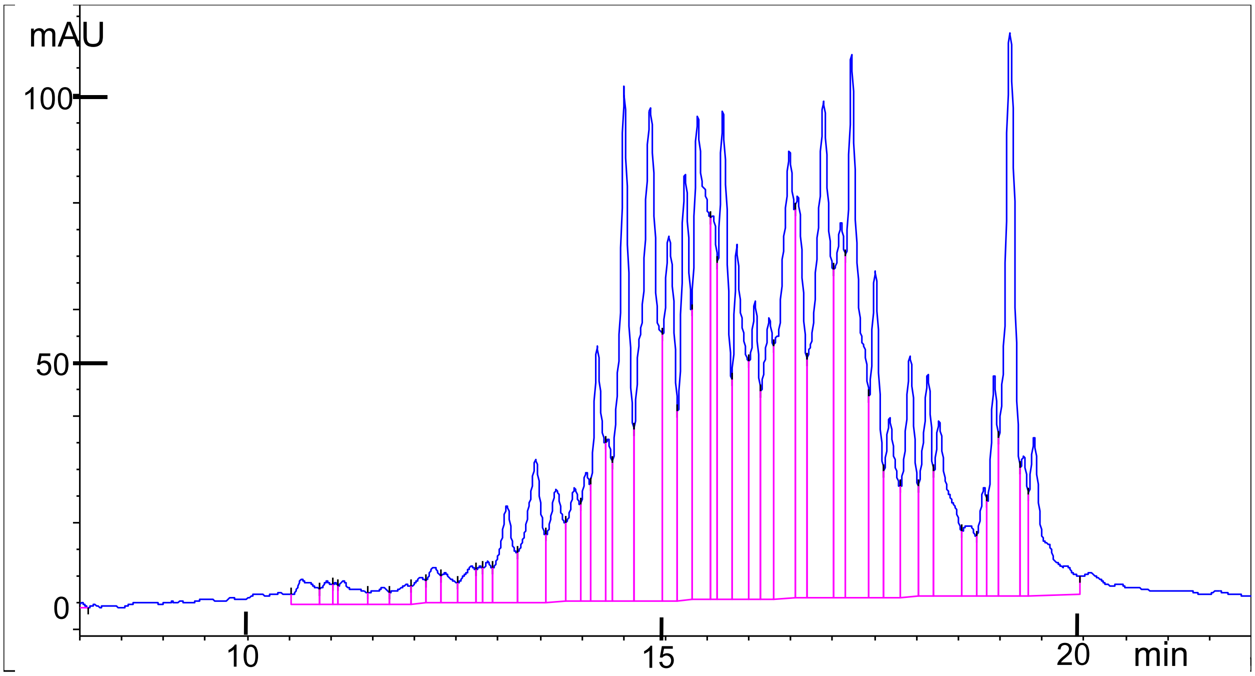

Figure S8: DNAPacIEX optimized method for tRNA E. Coli analysis at pH 12 and 10°C. Flow 0.8mL/min, 0.5mg/mL tRNA E.Coli from Sigma, in 25 min gradient from 40 to 70% of 1.5M NaCl in pH 12; 30uL injection volume, total area at 18,100 HPLC units, 48 peaks (separated by red lines) were automatically detected and integrated by the instrument.
